# 8-Chloroadenosine suppresses hepatocellular carcinoma progression via ADAR1/PPARγ axis-mediated lipid metabolism

**DOI:** 10.1016/j.gendis.2025.101874

**Published:** 2025-09-26

**Authors:** Jing Liu, Yongle Zhao, Xue Gong, Lin Yuan, Shengyong Yang, Wenwen Jian, Han Yan, Honglin Chen, Zhicheng Yang, Yiheng Sun, Tianle Gu, He Lu, Hongyun Zhao, Zeng Tu

**Affiliations:** a Department of Gastroenterology, The Second Affiliated Hospital of Chongqing Medical University, Chongqing 400016, China; b Department of Pathogen Biology, College of Basic Medical Science, Chongqing Medical University, Chongqing 400016, China; c Department of Laboratory Medicine, Binzhou Central Hospital, Binzhou, Shandong 251700, China; d Medical Laboratory Department, Xi'an No. 1 Hospital, Xi'an, Shaanxi 710000, China; e Department of Biochemistry and Molecular Biology, Molecular Medicine and Cancer Research Center, College of Basic Medicine, Chongqing Medical University, Chongqing 400016, China; f School of Savaid Stomatology, Hangzhou Medical College, Hangzhou, Zhejiang 311300, China; g The First Department of Internal Medicine, Changdu People's Hospital of Xizang, Xizang 854000, China

**Keywords:** 8-Chloroadenosine, ADAR1, Hepatocellular carcinoma, Lipid metabolism, PPARγ

## Abstract

The invasive and metastatic potential of hepatocellular carcinoma (HCC) is tightly linked to lipid metabolic reprogramming. However, existing knowledge of the molecular regulatory network governing lipid metabolism in HCC remains incomplete. This study reveals for the first time that ADAR1 promotes HCC progression via direct binding to *PPARγ* mRNA to regulate lipid metabolism. Through multi-omics approaches (publicly available single-cell sequencing databases, clinical sample analysis, and cellular models), we showed that ADAR1 was aberrantly up-regulated in HCC and promoted tumor cell proliferation, migration, and invasion. The adenosine analog 8-chloroadenosine (8-Cl-Ado) dose- and time-dependently down-regulated ADAR1 expression. Transcriptomic analysis revealed that 8-Cl-Ado significantly suppressed key genes associated with cholesterol synthesis and fatty acid metabolism. Mechanistically, ADAR1 binds to *PPARγ* mRNA, thereby activating the PPAR signaling axis, while *PPARγ* knockdown significantly abrogates malignant phenotypes in HCC. Functional rescue experiments confirmed that overexpression of the ADAR1 p150 isoform rescued the tumor-suppressive phenotype induced by 8-Cl-Ado. Collectively, these findings demonstrate that 8-Cl-Ado inhibits hepatocarcinogenesis and progression by suppressing ADAR1 and subsequently regulating PPARγ-mediated lipid metabolic processes, providing novel therapeutic targets and potential intervention strategies for HCC.

## Introduction

Primary liver cancer is a prevalent malignant tumor worldwide, ranking as the third leading cause of cancer-related mortality globally.^1^ Hepatocellular carcinoma (HCC) constitutes the predominant histological subtype of primary liver cancer, accounting for 75%–85% of cases.^2^^,^^3^ The high invasiveness and metastatic potential of HCC are key factors contributing to its propensity for recurrence and poor clinical outcomes.^4^^,^^5^ Metabolic reprogramming is recognized as a critical driver of malignant tumor progression,^6^, ^7^, ^8^ with aberrant lipid metabolism significantly promoting HCC development by remodeling the tumor microenvironment and activating oncogenic signaling pathways.^8^^,^^9^ However, the precise molecular mechanisms by which the lipid metabolic regulatory network contributes to tumorigenesis and progression remain incompletely elucidated, hindering the development of targeted therapeutic strategies. Therefore, a comprehensive dissection of lipid metabolism-related molecular mechanisms in HCC is crucial for elucidating its pathogenesis and identifying or validating potential therapeutic interventions.

The adenosine deaminase acting on RNA (ADAR) family regulates gene expression through its deaminase activity, catalyzing adenosine-to-inosine editing in double-stranded RNA, and by functioning as an RNA-binding protein.^10^, ^11^, ^12^ ADAR1 is ubiquitously expressed in nearly all tissues and exists as two isoforms, p110 and p150. ADAR1-mediated RNA editing, essential for mammalian survival, may contribute to carcinogenesis through an epigenetic mechanism.^11^, ^12^, ^13^ Notably, increased ADAR1 expression or activity has been associated with numerous cancers,^11^^,^^14^ including HCC,^15^ non-small cell lung cancer,^16^ gastric cancer,^17^ chronic myeloid leukemia,^18^ esophageal squamous cell carcinoma,^19^ colorectal cancer,^20^ oral squamous cell carcinoma,^21^ pancreatic cancer,^22^ multiple myeloma,^23^ cervical cancer,^24^ and thyroid cancer,^25^ suggesting its potential oncogenic role. Given the critical role of metabolic reprogramming, particularly lipid metabolism, in cancer progression, ADAR1 may exert its oncogenic functions by modulating metabolic pathways. However, this potential mechanism remains unclear in HCC.

8-Chloroadenosine (8-Cl-Ado), an adenine nucleoside analog, exhibits significant anti-tumor activity in various cell lines and animal models, including those for leukemia,^26^, ^27^, ^28^ cholangiocarcinoma,^29^ renal cell carcinoma,^30^ breast cancer,^31^ and multiple myeloma.^32^ Mechanistic studies suggest that 8-Cl-Ado can be sequentially phosphorylated by kinases in living cells to form 8-chloroadenosine triphosphate, which suppresses cell proliferation by interfering with RNA synthesis and energy consumption in the ATP pool. Additionally, 8-Cl-Ado is metabolized to an adenylosuccinate analog, linking its metabolism to the tricarboxylic acid cycle by reducing the fumarate pools.^33^^,^^34^ These findings indicate that 8-Cl-Ado has multi-target effects. Given that ADAR1 acts on adenosine residues in RNA and 8-Cl-Ado is an adenine nucleoside analog, we hypothesize that ADAR1 is modulated by 8-Cl-Ado to exert its anti-HCC effects.

Dysregulated lipid metabolism is significantly associated with HCC risk.^8^^,^^9^ Peroxisome proliferator-activated receptors (PPARs) are ligand-activated transcription factors belonging to the nuclear receptor superfamily. Comprising three subtypes (*PPARα*, *PPARδ*, and *PPARγ*), they play broad roles in physiological processes, including lipid metabolism, glucose homeostasis, and inflammation.^35^^,^^36^ Lipids serve as endogenous ligands for all *PPAR*s, directly linking them to metabolism.^35^ Among the subtypes, *PPARγ* plays a more prominent role in adipogenesis and lipid synthesis. As a key regulator of lipid metabolism, *PPARγ* influences cancer cell growth, migration, and invasion by transcriptionally activating or repressing processes, such as fatty acid uptake, fatty acid synthesis, and cholesterol synthesis.^37^^,^^38^ Studies have shown that *PPARγ* activation promotes glycolysis in liver cancer cells, thereby enhancing HCC proliferation, migration, and invasion.^39^ Consequently, *PPARγ* is considered a potential molecular target for HCC intervention. However, it remains unknown whether and how ADAR1 affects HCC lipid metabolism and the growth, migration, and invasion of liver cancer cells via regulation of the *PPARγ* signaling pathway.

This study aims to elucidate the role of ADAR1 in lipid metabolism in HCC and its effects on tumor cell growth, migration, and invasion, while exploring whether the anti-tumor mechanism of 8-Cl-Ado involves this pathway. Our results demonstrate that 8-Cl-Ado effectively suppresses the proliferation, migration, and invasion of HCC cells (HepG2, Huh7). Mechanistically, we confirmed ADAR1's involvement in the anti-cancer effects of 8-Cl-Ado. Transcriptomic analysis revealed that 8-Cl-Ado modulated PPARγ-mediated lipid metabolic processes, thereby influencing HCC cell growth. Crucially, RNA immunoprecipitation experiments confirmed a binding interaction between ADAR1 and *PPARγ* mRNA. In summary, this study demonstrates for the first time that 8-Cl-Ado suppresses HCC tumorigenesis and progression by inhibiting *ADAR1*, subsequently regulating the *PPARγ* signaling pathway and its mediated lipid metabolic processes. These findings provide novel mechanistic insights and potential intervention strategies for HCC treatment.

## Materials and methods

### Cell culture

HepG2 cells and Huh7 cells were cultured in Dulbecco's Modified Eagle's Medium (Gibco), supplemented with 10% fetal bovine serum (ExCell) and 1% penicillin/streptomycin (HyClone). The cells were grown in a humidified environment with 5% carbon dioxide at 37 °C. Cells were passaged when they reached 90% confluence.

### Chemicals and reagents

8-Cl-Ado (CAS.NO 34408-14-5) with a purity of ≥ 99% was purchased from R&D Systems. Palmitate was purchased from Sigma and dissolved in 1% bovine serum albumin (Shanghai). Rat tail collagen type I (attailtendon collagen type I, Solarbio) was used to coat the surface of cell culture dishes.

### Cell transfection

For small interfering RNAs (siRNAs), cells were transfected with siRNAs specific for *ADAR1* or *PPARγ* with Lipofectamine 3000 for 6 h according to the manufacturer's instructions. The cells were then replaced with normal medium and used for subsequent experiments 48 h after infection. For ADAR1 overexpression studies, cells were seeded in 6-well plates at 70% confluency and transfected with 2 μg of ADAR1 p150 or p110 expression plasmid (or relevant plasmid source) using Lipofectamine 3000 according to the manufacturer's protocol. Following 48 h of incubation, transfected cells were harvested for downstream analyses. The sequences of *ADAR1* or *PPARγ*-specific siRNA are shown in Table 1.

**Table 1:** Nucleotide sequences of the sense and the antisense strands of *hADAR siRNA* and *hPPARγ siRNA*.

Name	Species	Sense	Antisense
*ADAR* si-1	Human	5′-GCCCACUGUUAUCUUCACUUUTT-3′	5′-AAAGUGAAGAUAACAGUGGGCTT-3′
*ADAR* si-2	Human	5′-GCUGUUAGAAUAUGCCCAGUUTT-3′	5′-AACUGGGCAUAUUCUAACAGCTT-3′
*ADAR* si-3	Human	5′-GAGGAUGCAAAUCAAGAGAAATT-3′	5′-UUUCUCUUGAUUUGCAUCCUCTT-3′
*PPARγ* si-1	Human	5′-CAGCAUUUCUACUCCACAUUATT-3′	5′-UAAUGUGGAGUAGAAAUGCUGTT-3′
*PPARγ* si-2	Huamn	5′-UCCGUGGAUCUCUCCGUAAUGTT-3′	5′-CAUUACGGAGAGAUCCACGGATT-3′
*PPARγ* si-3	Human	5′-GACAACAGACAAAUCACCAUUTT-3′	5′-AAUGGUGAUUUGUCUGUUGUCTT-3′

For ADAR1 overexpression and 8-Cl-Ado treatment, cells were seeded in 6-well plates at 70% confluency. After 24 h of adherence, cells were transfected with 2 μg of ADAR1 overexpression plasmid (or pCMV empty vector control). At 24 h post-transfection, cells were treated with 8-Cl-Ado (10 μM) for an additional 24 h before subsequent functional assays.

### Cell viability assay

The proliferative capacity of HepG2 cells and Huh7 cells was assayed using Cell Counting Kit-8 (CCK-8). For the CCK-8 assay, HepG2 cells and Huh7 cells were cultured in humidified gas containing 5% CO_2_ at a density of 8000 cells per well in 96-well plates at 37 °C. After appropriate treatments, 10 μL of CCK-8 solution was added to each well and then incubated for 1 h. The absorbance at 450 nm of each well was measured using an enzyme marker (Bio-Rad, Hercules, California, USA).

### Migration assay

HepG2 cells and Huh7 cells were seeded in 6-well plates and grown up to 95% confluency, and a linear wound in the cell monolayer was created using a 200 μL tip, followed by washing with phosphate buffer saline to eliminate detached cells. The experiment involved performing different treatments for specific purposes. Cell migration was estimated by measuring the distance travelled using a microscope to take photographs.

### Transwell migration and Matrigel-based invasion assays

Cell migration and invasion tests were performed using Transwell chambers (Corning, USA). In brief, 200 μL of the HepG2 cells and Huh7 cells (1 × 10^4^) were loaded on the upper chamber after 24 h of serum-free starvation. Subsequently, the lower chamber was supplemented with 600 μL of 20% fetal bovine serum-supplemented Dulbecco's Modified Eagle's Medium, and kept in an incubator at a consistent temperature of 37 °C and 5% CO_2_ for 48 h. After incubation, cells attached to the bottom of the membrane were fixed using 4% paraformaldehyde and stained with 0.2% crystal violet. Finally, invading cells were counted under a phase-contrast microscope. Cell migration and invasion assays shared the same protocols, except for the presence of Matrigel in the upper chambers (Corning, USA) for the invasion assays.

### RNA isolation and sequencing

HepG2 cells and Huh7 cells were treated with 0, 10 μmol/L 8-Cl-Ado for 24 h. Total cellular RNA was extracted using TRIzol reagent (Invitrogen) according to the manufacturer's instructions and sequenced by Majorbio Co., Ltd. (Shanghai, China) using Illumina NovaSeq 6000. RNA concentration and purity were measured using Nodrop2000, and RNA integrity was determined using Agilent 5300.

### Real-time fluorescence quantitative PCR

Total RNA was extracted using TRIzol reagent (Invitrogen). cDNA was synthesized using the PrimeScript™ RT Reagent Kit with gDNA Eraser (Takara) according to the manufacturer's protocol. Real-time quantitative PCR was performed using the 2 × Universal SYBR Green Fast qRT-PCR Mix (Abclonal) with the following reaction conditions: 1 cycle of 95 °C for 3 min, 40 cycles of 95 °C for 5 s, and 60 °C for 30 s. All PCR primers were designed using Primer-BLAST (NCBI) (Table 2) and were synthesized by Beijing Tsingke Biotech Co., Ltd (China). Target gene expression was normalized to the expression of the *GAPDH* gene, and relative expression was calculated using the 2^−ΔΔCt^ method.

**Table 2:** Nucleotide sequences of primers used for quantitative PCR.

Gene	Species	Forward primer sequence	Reverse primer sequence
*PPARα*	Human	5′-CTATAATTTGCTGTGGAGATCGGC-3′	5′-GGATGGTTGCTCTGCAGGT-3′
*PPARγ*	Human	5′-ACCAAAGTGCAATCAAAGTGGA-3′	5′-ATGAGGGAGTTGGAAGGCTCT-3′
*PPARδ*	Human	5′-CGGACCTGGGGATTAATGGG-3′	5′-CAGACCATTCCAGACCCTCG-3′
*ADAR1* p110	Human	5′-GGCAGCCTCCGGGTG-3′	5′-CTGTCTGTGCTCATAGCCTTGA-3′
*ADAR1* p150	Human	5′-CGGGCAATGCCTCGC-3′	5′-AATGGATGGGTGTAGTATCCGC-3′
*MPC1*	Human	5′-GTCATTGGCTCTGGGAAGCG-3′	5′-GGCCCCAGAAGTGCGTA-3′
*PDK1*	Human	5′-CATGTCACGCTGGGTAATGAGG-3′	5′-CTCAACGAGGTCTTGGTGCA-3′
*SLC25A1*	Human	5′-GCCGTCAGGTTTGGAATGTT-3′	5′-GAGGTCTGGTCGTGGATGAA-3′
*FASN*	Human	5′-GAGACACTCGTGGGCTACAG-3′	5′-CTCAAGAACTGCACGGAGGT-3′
*ELOVL6*	Human	5′-GGATGCAGGAAAACTGGAAG-3′	5′-ATTCATTAGGTGCCGACCAC-3′
*SCD*	Human	5′-TTCCCGACGTGGCTTTTTCT-3′	5′-AAGCCAGGTTTGTAGTACCTCC-3′
*ACSS1*	Human	5′-CGTCCTTTTTGAGAGCACCC-3′	5′-GCATCACCGTATTTCAGCAACA-3′
*DGAT1*	Human	5′-AACTACCGTGGCATCCTGAA-3′	5′-AGAGAAACCACCTGGATGGG-3′
*FABP1*	Human	5′-ATCAAGGGGGTGTCGGAAAT-3′	5′-CCAACTGAACCACTGTCTTGACT-3′
*FABP5*	Human	5′-AAAACTGAGAGCACTTTGAAAACAAC-3′	5′-TTTCTGCCATCAGCTGTGGTT-3′
*HMGCS1*	Human	5′-GATGTGGGAATTGTTGCCCTT-3′	5′-ATTGTCTCTGTTCCAACTTCCAG-3′
*HMGCR*	Human	5′-TACCATGTCAGGGGTACGTC-3′	5′-CAAGCCTAGAGACATAAT-3′
*MVD*	Human	5′-GTGACCTCTCAGAAGTGGCT-3′	5′-CTCACCACAAGGATGAGCAC-3′
*FDPS*	Human	5′-AGGGCAATGTGGATCTTGTC-3′	5′-GAAAGAACTCCCCCATCTCC-3′
*IDI1*	Human	5′-ATGGAGCAAGTCTGTCAGCA-3′	5′-GCTCGATGCAATAATCCTTTCTC-3′
*FDFT1*	Human	5′-GGCAGTACCTGACCACTCTC-3′	5′-TCCTAAAGGTCCCAGCCACA-3′
*ERLIN1*	Human	5′-ATCCACCATGAGCTGAACCA-3′	5′-ACGCACAGCCTGTATAGTGA-3′
*SQLE*	Human	5′-TCCTTGCTCAGGCTCTTTATG-3′	5′-AGGGTTAGGAGACAATACAGAAAG-3′
*LSS*	Human	5′-ATCTCTGGATGGGCCTTGAC-3′	5′-TCTGGTAGTCGGGAGGGTTA-3′
*DHCR7*	Human	5′-TTCTCGCCCACCATCATCTT-3′	5′-CGAGGGTTAAACTCGATGCC-3′

### Western blotting

Total proteins were separated by RIPA lysate buffer (Beyotime Biotechnology) and quantified by the bis-succinic acid method. An amount of 10 μg of each protein sample was collected, separated by SDS-polyacrylamide gel electrophoresis, and transferred to a PVDF membrane (0.45 μm). The membrane was then blocked with 5% skimmed milk and incubated with ADAR1 (CY8635; 1:1000; ABWAYS), PPARα (66826-1-Ig; 1:2000; Proteintech, USA), PPARδ (60193-1-Ig; 1:2000; Proteintech, USA), PPARγ (16643-1-AP; 1:2000; Proteintech, USA), and β-actin (AF2815; 1:5000; BIOTEN BioTechnology Inc) primary antibodies at 4 °C overnight. The membrane was then washed three times with Tris-buffered saline-Tween20 buffer and incubated with horseradish peroxidase-conjugated secondary antibody (1:5000; Proteintech, USA) at room temperature for 1 h. The membranes were washed three times and then immunodetected using an enhanced chemiluminescence kit (Beyotime Biotechnology).

### Oil red O staining

Sterilized cell culture coverslips were placed in Petri dishes before plating the cells to facilitate observation of the staining. Staining was carried out following the instructions for oil red O staining reagent (Sigma, O0625). Cells were mounted on slides and observed under a microscope after staining. Lipids were quantified using isopropanol. To destain the lipid droplets, 60% isopropanol was added immediately after oil red O staining, and the lipid droplets were extracted in the dark at room temperature for 20 min. The absorbance at OD450 nm was measured using a microplate reader.

### Investigation of triglycerides and cholesterol

Cells were treated as previously described. Briefly, 1 × 10^6^ cells were diluted into 200 μL of phosphate buffer saline, followed by ultrasonic trituration to obtain cellular homogenate. The levels of cellular triglycerides and total cholesterol were investigated according to the manufacturer's instructions (Cat. No. 000180 and 000220; Nanjing Jiancheng Bioengineering Institute). Cellular homogenates were normalized to total protein content as detected by the bis-succinic acid assay.

### RNA immunoprecipitation

HEK293T cells transfected with *ADAR1 p150* plasmid were lysed in pre-chilled 1% NP-40 lysis buffer (150 mM NaCl, 50 mM Tris–HCl, pH 8.0, 10% NP-40) supplemented with protease inhibitors. Lysates were centrifuged (12,000 rpm, 4 °C) for 20 min, and the supernatants were then collected. Input samples (10% total lysate) were reserved for RNA isolation. The remaining lysate was divided equally and incubated at 4 °C overnight with 2 μg of anti-ADAR1 antibody (Proteintech) or control IgG. Protein A/G magnetic beads (Thermo Fisher) were washed with lysis buffer and added to the antibody-lysate mixtures, followed by rotation for 4–6 h at 4 °C. Beads were pelleted magnetically and washed four times with lysis buffer. RNA was extracted from immunoprecipitates and Input samples using TRIzol reagent (Invitrogen) according to the manufacturer's protocol. Purified RNA was reverse-transcribed into cDNA, and *PPARγ* mRNA enrichment was quantified by real-time quantitative PCR using *PPARγ*-specific primers. Data were normalized to the input samples.

### Transcriptomic data processing and differential gene expression analysis

RNA sequencing was performed on HepG2 cells treated with 10 μM 8-Cl-Ado and untreated controls (three biological replicates per group). After quality control (removal of low-quality reads), clean reads were aligned to the human reference genome (GRCh38) using HISAT2. Gene expression quantification was conducted via transcripts per million (TPM) normalization. Differentially expressed genes were identified using DESeq2, with thresholds set at |log_2_(fold change)| > 1 and adjusted *p*-value <0.05.

### Functional enrichment analyses

Venn diagrams (https://jvenn.toulouse.inra.fr/app/index.html) were employed to identify identical differentially expressed genes between HepG2 and Huh7 cells treated with 8-Cl-Ado (*p* < 0.05), which were subsequently subjected to enrichment analyses. Kyoto Encyclopedia of Genes and Genomes (KEGG) pathway enrichment was analyzed using KOBAS (v3.0) and Fisher's exact test, applying the same significance threshold. The top 20 GO terms and top 10 KEGG pathways ranked by enrichment significance were selected for downstream validation and interpretation. Heatmap analysis was subsequently conducted for the 44 genes comprising the top 10 enriched KEGG pathways, visualizing their Log_2_(fold change) values derived from the RNA-sequencing data of both cell lines. Additionally, a separate heatmap analysis was performed for 20 lipid metabolism-related genes associated with fatty acids and cholesterol pathways, using the same Log_2_(fold change) metric. All analyses utilized default parameters unless otherwise specified.

### Bioinformatic analysis of clinical data using GEPIA

*ADAR1* expression in HCC was analyzed using the Gene Expression Profiling Interactive Analysis (GEPIA, http://gepia.cancer-pku.cn/) platform. RNA-sequencing data from 369 HCC tumors (TCGA) and 160 normal liver tissues (GTEx) were processed to evaluate differential ADAR1 expression. Associations between ADAR1 levels and HCC overall survival were further assessed.

### Statistical analysis

In each experiment, each condition or treatment was performed in at least triplicate. GraphPad Prism 10.5.0 (GraphPad Prism, San Diego, California, USA) was applied for statistical analysis and plotting, with data expressed as mean ± standard error of the mean. Student's *t*-test (two-tailed) was used to compare two groups, and one-way ANOVA was used to compare multiple groups. *P-*values < 0.05 were considered statistically significant (^∗^*p* < 0.05, ^∗∗^*p* < 0.01, and ^∗∗∗^*p* < 0.001).

## Results

### ADAR1 is aberrantly overexpressed in liver cancer tissues

**Figure 1:**
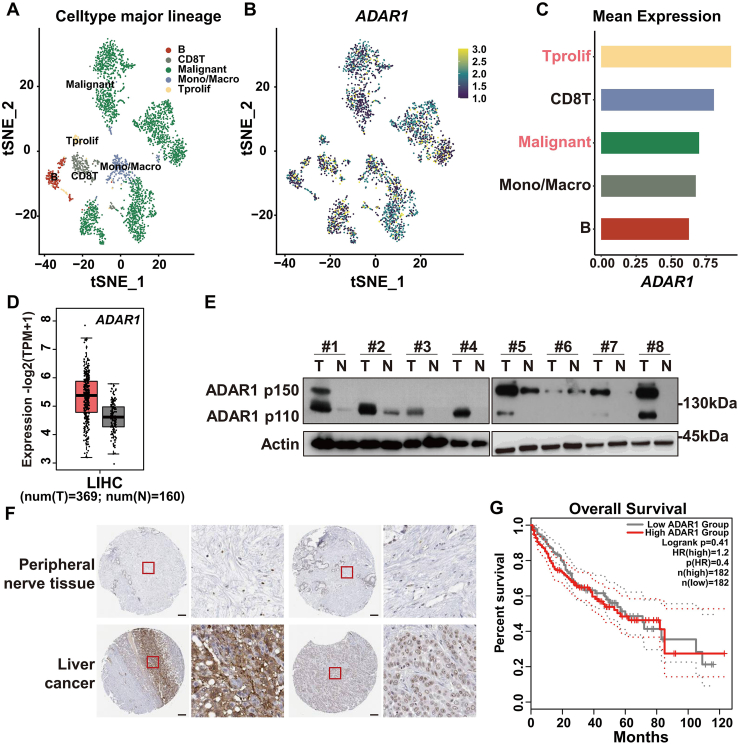
**ADAR1 is aberrantly overexpressed in liver cancer tissues and associated with poor prognosis. (A**–**C)** Analysis of *ADAR1* expression abundance across human cell lineages by single-cell RNA sequencing. (A) Cell lineage markers. (B) Expression pattern of *ADAR1* across distinct cell populations. (C) Median expression levels of *ADAR1* in different cell clusters. **(D)** Analysis of the GEPIA database demonstrating relative *ADAR1* levels in hepatocellular carcinoma (HCC) tumor tissues (red box, *n* = 369) versus normal tissues (gray box, *n* = 160). **(E)** ADAR1 protein expression levels in paired clinical HCC tumor tissues (T) and adjacent non-tumor tissues (N) (*n* = 8). This experiment was performed with at least three biologically independent replicates (*n* ≥ 3). **(F)** Representative immunohistochemical staining images from the HPA database. Scale bar: 100 μm. **(G)** Overall survival analysis of HCC patients stratified by *ADAR1* expression levels in the GEPIA database.

Single-cell sequencing data analysis revealed the highest expression abundance of *ADAR1* within the abnormally proliferating tumor cell population (Tprolif) (Fig. 1A–C). Analysis of the GEPIA database demonstrated significantly elevated *ADAR1* mRNA expression levels in HCC tissues compared with normal tissues (Fig. 1D). Western blot analysis of eight paired clinical HCC and matched adjacent non-tumor tissues further confirmed significantly increased protein expression of both ADAR1 p150 and p110 isoforms in tumor tissues (Fig. 1E). Data from the Human Protein Atlas also supported the high expression of *ADAR1* in liver cancer tissues (Fig. 1F). Survival analysis indicated that patients with high *ADAR1* expression exhibited moderately shorter overall survival times compared with those with low expression, although this difference was not statistically significant (Fig. 1G).

### ADAR1 promotes the proliferation, migration, and invasion of liver cancer cells

**Figure 2:**
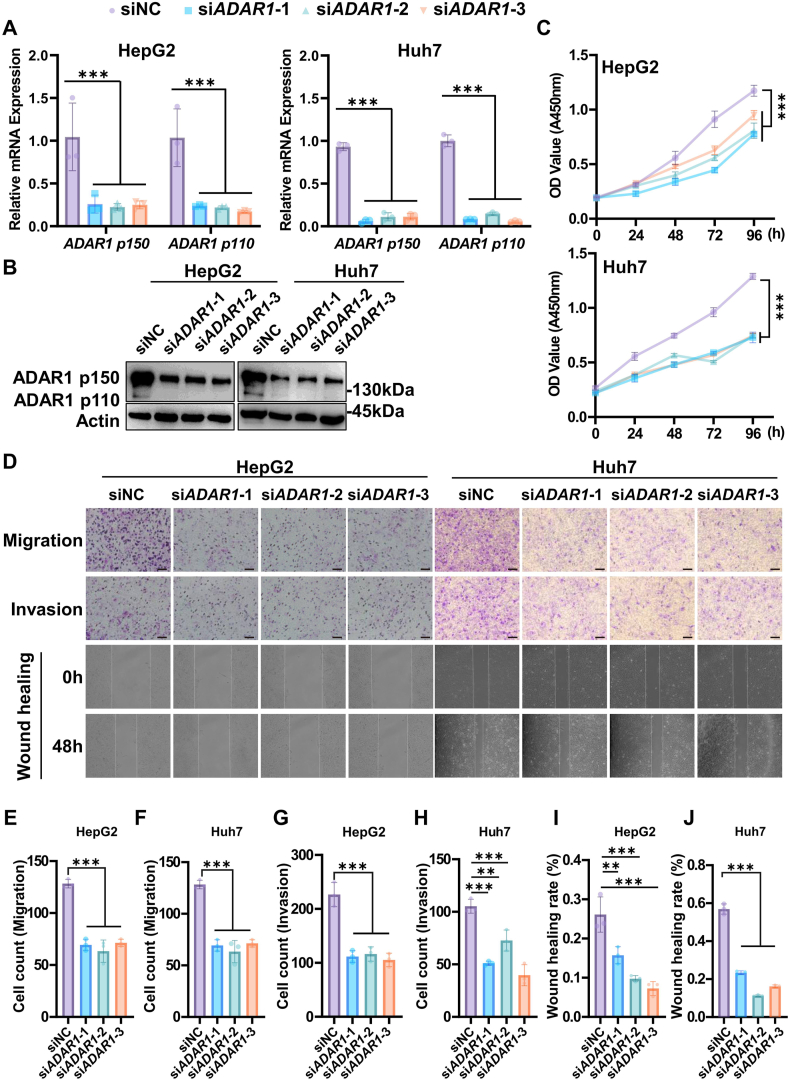
**Knockdown of *ADAR1* suppresses proliferation, migration, and invasion of liver cancer cells. (A)***ADAR1* mRNA expression levels in HepG2 and Huh7 cells following *ADAR1* knockdown. **(B)** ADAR1 protein expression levels in HepG2 and Huh7 cells following *ADAR1* knockdown. **(C)** Effect of *ADAR1* knockdown on OD450 nm absorbance in HepG2 and Huh7 cells with CCK-8 assay. **(D)** Representative images showing the impact of *ADAR1* knockdown on the migration, invasion, and wound healing capacities of HepG2 and Huh7 cells. **(E)** Quantification of *ADAR1* knockdown effects on HepG2 cell migration. Scale bar: 20 μm. **(F)** Quantification of *ADAR1* knockdown effects on Huh7 cell migration. **(G)** Quantification of *ADAR1* knockdown effects on HepG2 cell invasion. **(H)** Quantification of *ADAR1* knockdown effects on Huh7 cell invasion. **(I)** Quantification of *ADAR1* knockdown effects on HepG2 cell wound healing capacity. **(J)** Quantification of *ADAR1* knockdown effects on Huh7 cell wound healing capacity. All experiments were performed with at least three biologically independent replicates (*n* ≥ 3). Data were presented as mean ± standard error of the mean. Statistical significance was determined using Student's two-tailed *t*-test for two-group comparisons or one-way ANOVA for multiple groups. ^∗∗^*p* < 0.01, ^∗∗∗^*p* < 0.001.

To further investigate the role of ADAR1 in liver cancer progression, we performed loss-of-function and gain-of-function experiments: ADAR1 expression was knocked down using siRNAs targeting *ADAR1* in HepG2 and Huh7 cells (Fig. 2A and B), and ADAR1 p150/p110 isoforms were overexpressed using pCMV-driven expression plasmids (Fig. S1A–S1D). Cell proliferation assays demonstrated that *ADAR1* knockdown (si*ADAR1* groups) significantly inhibited cell proliferation (Fig. 2C), while ADAR1 overexpression enhanced proliferation (Fig. S2E). Transwell assays showed that *ADAR1* knockdown impaired cell migration and invasion capabilities (Fig. 2D–H), whereas ADAR1 overexpression enhanced these abilities in HCC cells (Fig. S2A–S2C). Similarly, *ADAR1* knockdown significantly suppressed cell wound repair capacity (Fig. 2D–I, J), and *ADAR1* overexpression enhanced wound repair capacity (Fig. S2A and S2D). Collectively, these results indicate that ADAR1 promotes proliferation, migration, and invasion of HCC cells.

#### *Cl-Ado suppresses liver cancer progression by reducing ADAR1 expression*

**Figure 3:**
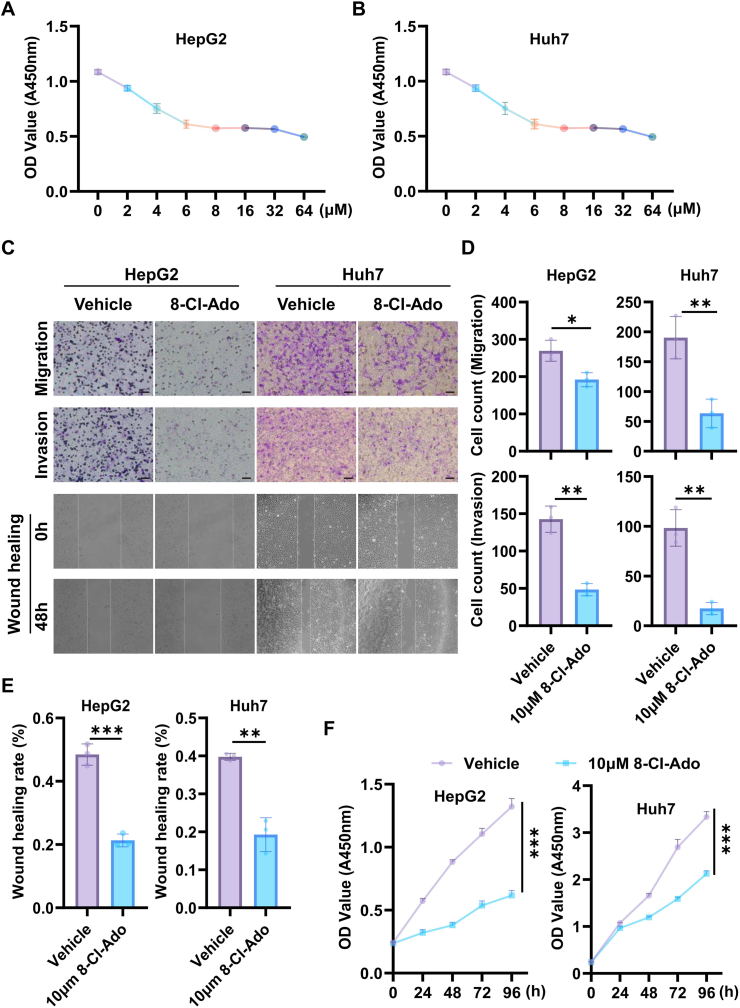
**8-Cl-Ado suppresses liver cancer progression. (A)** Effect of varying concentrations of 8-Cl-Ado on OD450 nm absorbance in HepG2 cells with CCK-8 assay. **(B)** Effect of varying concentrations of 8-Cl-Ado on OD_450 nm_ in Huh7 cells with CCK-8 assay. **(C)** Representative images showing the impact of 8-Cl-Ado on migration, invasion, and wound healing capacities of HepG2 and Huh7 cells. Scale bar: 20 μm. **(D)** Quantification of 8-Cl-Ado effects on migration and invasion capabilities in HepG2 and Huh7 cells. **(E)** Quantification of 8-Cl-Ado effects on wound healing capacity in HepG2 and Huh7 cells. **(F)** Effect of 8-Cl-Ado on OD_450_ in HepG2 and Huh7 cells with CCK-8 assay. All experiments were performed with at least three biologically independent replicates (*n* ≥ 3). Data were presented as mean ± standard error of the mean. Statistical significance was determined using Student's two-tailed *t*-test for two-group comparisons or one-way ANOVA for multiple groups. ^∗^*p* < 0.05, ^∗∗^*p* < 0.01, ^∗∗∗^*p* < 0.001.

As an adenine nucleoside analog, 8-Cl-Ado exhibits significant anti-cancer activity.^27^^,^^29^^,^^40^ Its effects were investigated in HepG2 and Huh7 cells. Initial evaluation in HepG2 and Huh7 cells yielded 24-h half-maximal inhibitory concentrations (IC_50_) of approximately 10 μmol/L for both cell lines (Fig. 3A and B). Concurrently, 8-Cl-Ado treatment effectively inhibited cell migration, invasion, wound repair, and proliferation capabilities (Fig. 3C–F).

**Figure 4:**
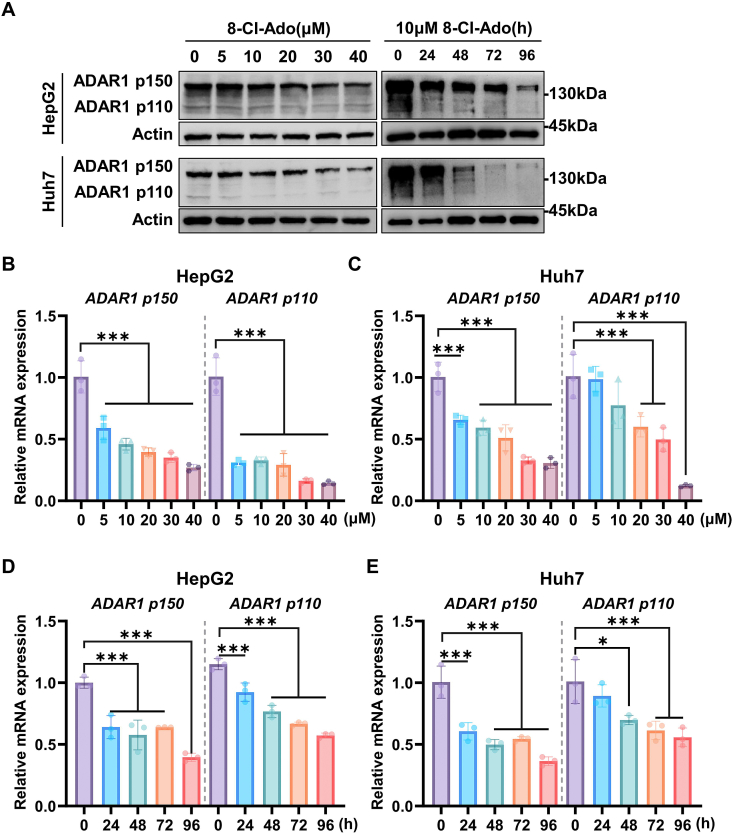
**8-Cl-Ado induces a dose- and time-dependent decrease in ADAR1 expression. (A)** Effect of 8-Cl-Ado at varying concentrations and treatment durations on ADAR1 protein expression levels in HepG2 and Huh7 cells. **(B)** Effect of different 8-Cl-Ado concentrations on *ADAR1* mRNA expression in HepG2 cells. **(C)** Effect of different 8-Cl-Ado concentrations on *ADAR1* mRNA expression in Huh7 cells. **(D)** Effect of different 8-Cl-Ado treatment durations on *ADAR1* mRNA expression in HepG2 cells. **(E)** Effect of different 8-Cl-Ado treatment durations on *ADAR1* mRNA expression in Huh7 cells. All experiments were performed with at least three biologically independent replicates (*n* ≥ 3). Data were presented as mean ± standard error of the mean. Statistical significance was determined using Student's two-tailed *t*-test for two-group comparisons or one-way ANOVA for multiple groups. ^∗^*p* < 0.05, ^∗∗∗^*p* < 0.001.

Given ADAR1's strong nucleotide dependence, the effects of 8-Cl-Ado on ADAR1 were further examined using varying concentrations and treatment durations. Results demonstrated that 8-Cl-Ado reduced both protein (Fig. 4A) and mRNA (Fig. 4B–E) expression levels of ADAR1 in a time- and dose-dependent manner in HepG2 and Huh7 cells. These findings suggest that 8-Cl-Ado inhibits HCC cell proliferation, migration, and invasion, at least partially, by suppressing ADAR1 transcription and translation.

**Figure 5:**
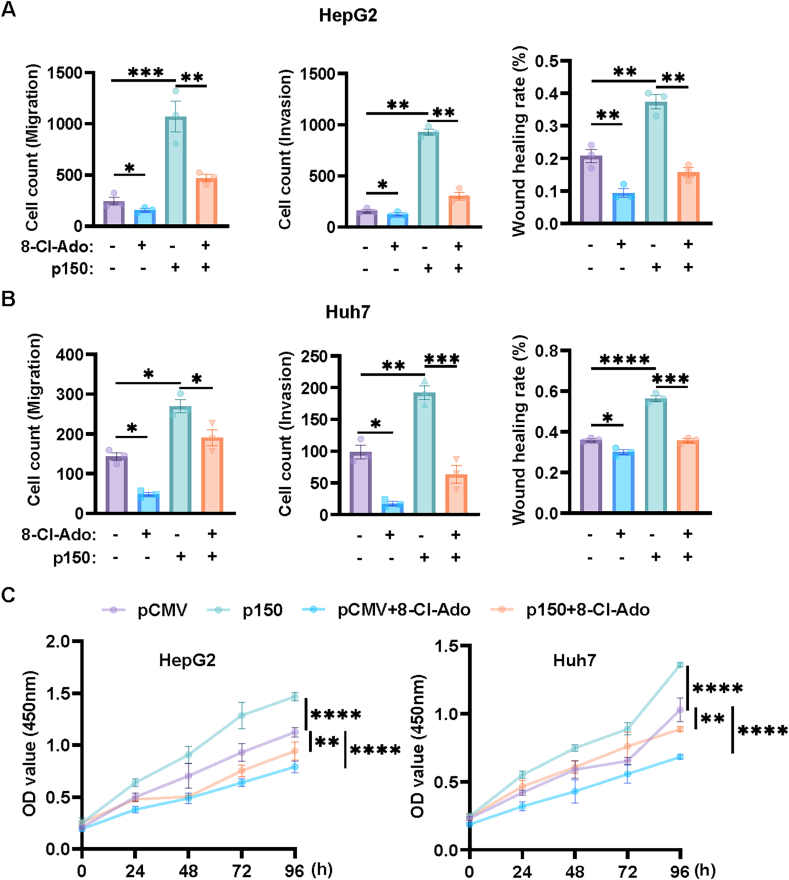
**ADAR1 overexpression partially counteracts 8-Cl-Ado-mediated inhibition of liver cancer progression. (A)** Effects of 8-Cl-Ado treatment modulation via ADAR1 overexpression on migration, invasion, and wound healing capacities of HepG2 cells. **(B)** Effects of 8-Cl-Ado treatment modulation via ADAR1 overexpression on migration, invasion, and wound healing capacities of Huh7 cells. **(C)** Effects of 8-Cl-Ado treatment modulation via ADAR1 overexpression on OD_450_ in HepG2 and Huh7 cells with CCK-8 assay. All experiments were performed with at least three biologically independent replicates (*n* ≥ 3). Data were presented as mean ± standard error of the mean. Statistical significance was determined using Student's two-tailed *t*-test for two-group comparisons or one-way ANOVA for multiple groups. ^∗^*p* < 0.05, ^∗∗^*p* < 0.01, ^∗∗∗^*p* < 0.001.

To validate whether ADAR1 mediated 8-Cl-Ado's anti-cancer effects, functional rescue experiments were performed. Cells co-treated with ADAR1 p150 overexpression and 8-Cl-Ado exhibited intermediate migration, invasion, wound repair, and proliferation capabilities compared with the ADAR1 overexpression-only and 8-Cl-Ado treatment-only groups (Fig. 5A–C; Fig. S3A and S3B), indicating that ADAR1 p150 overexpression counteracts 8-Cl-Ado-mediated inhibition.

In summary, ADAR1 p150 overexpression markedly attenuated the inhibitory effects of 8-Cl-Ado on HCC cell proliferation, migration, and invasion, suggesting *ADAR1* is a key molecular target for 8-Cl-Ado's anti-HCC activity.

### Transcriptomics identifies 8-Cl-Ado suppresses HCC development via lipid metabolism-related gene regulation

**Figure 6:**
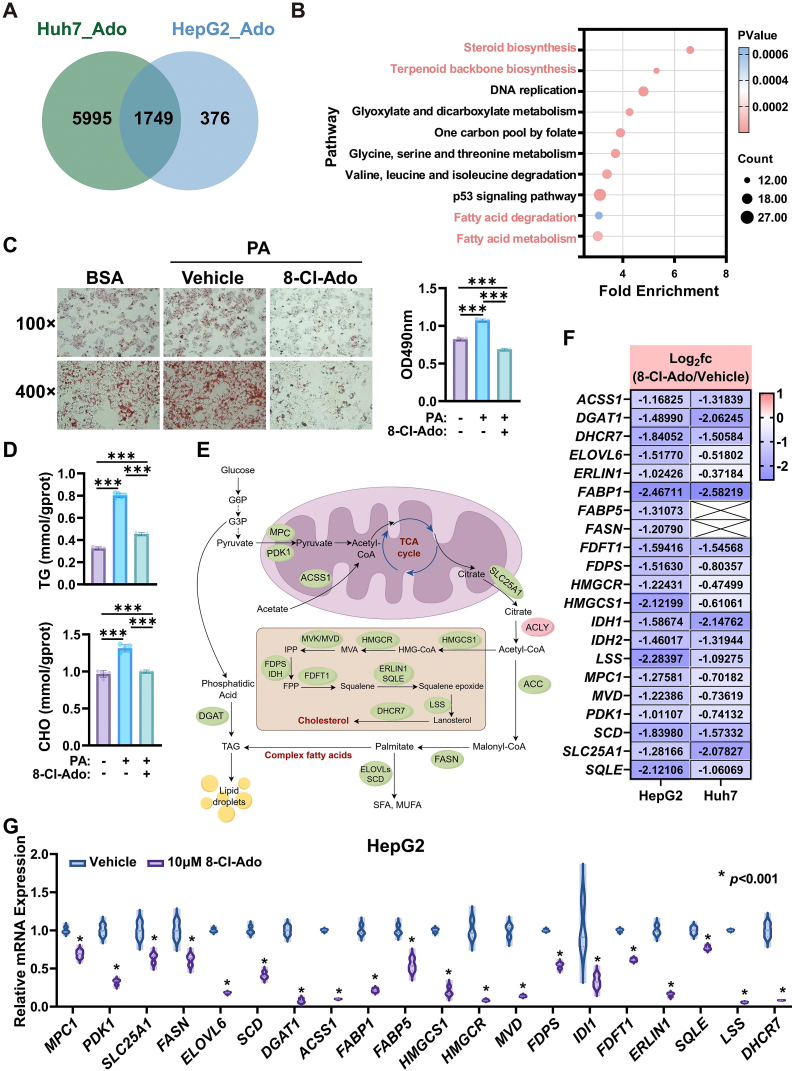
**8-Cl-Ado suppresses lipid metabolism in liver cancer by regulating gene expression. (A)** The Venn diagram showing common differentially expressed genes (DEGs, *p* < 0.05) in HepG2 and Huh7 cells treated with 8-Cl-Ado for 24 h. **(B)** KEGG pathway enrichment analysis of common DEGs (top 10 pathways; 4 lipid metabolism-related pathways highlighted in red). **(C)** Effect of 8-Cl-Ado on lipid accumulation in HepG2 cells (Left: Representative images of oil red O staining; Right: Quantification of lipid droplet detection by oil red O staining). **(D)** Effect of 8-Cl-Ado on intracellular total cholesterol (CHO) and triglyceride (TG) levels in liver cancer cells. **(E)** Schematic diagram of CHO and fatty acid biosynthesis pathways. **(F)** The heatmap visualizing the expression patterns and significance (Log_2_(fold change)) of key genes involved in CHO biosynthesis and fatty acid metabolism (*n* = 20) in 8-Cl-Ado-treated HepG2 and Huh7 cells. **(G)** Effect of 8-Cl-Ado on the expression levels of key genes involved in CHO biosynthesis and fatty acid metabolism in HepG2 cells (*n* = 20). All experiments were performed with at least three biologically independent replicates (*n* ≥ 3). Data were presented as mean ± standard error of the mean. Statistical significance was determined using Student's two-tailed *t*-test for two-group comparisons or one-way ANOVA for multiple groups. ^∗∗∗^*p* < 0.001.

Venn analysis of differentially expressed genes with a *p*-value < 0.05 in 8-Cl-Ado-treated HepG2 and Huh7 cells identified 1749 common genes (Fig. 6A). Enrichment analysis revealed that these genes were significantly enriched in steroid biosynthesis, fatty acid metabolism, and related pathways (Fig. 6B). These findings suggest that 8-Cl-Ado may suppress HCC progression by modulating the expression of lipid metabolism-associated genes (Fig. S3C). To further validate these findings, an *in vitro* lipid accumulation model induced by palmitate was established. 8-Cl-Ado treatment significantly reduced palmitate-induced lipid droplet accumulation (Fig. 6C) and decreased intracellular triglyceride and total cholesterol levels (Fig. 6D).

Based on the specific molecular mechanisms of cholesterol and fatty acid biosynthesis,^9^^,^^37^^,^^41^ focus was placed on key genes involved in these processes (Fig. 6E). RNA sequencing revealed that 8-Cl-Ado treatment significantly down-regulated key genes involved in fatty acid uptake, synthesis, and esterification (*HMGCS1*, *HMGCR*, *MVD*, *FDPS*, *IDH*, *FDFT1*, *SQLE*, *ERLIN1*, *LSS*, and *DHCR7*) and cholesterol synthesis/regulation (*ACSS1*, *FASN*, *SCD*, *ELOVL6*, *DAGT1*, *SLC25A1*, *PDK1*, and *MPC1*) (Fig. 6F). Real-time quantitative PCR validation confirmed these transcriptional changes showing consistent down-regulation (Fig. 6G). These results validate the reliability of the transcriptomic data and demonstrate that 8-Cl-Ado can inhibit lipid metabolic activity in HCC cells.

### PPARγ mediates the regulatory effects of 8-Cl-Ado on lipid metabolism

To validate the transcriptomics results and further investigate 8-Cl-Ado's regulation of lipid metabolism, KEGG pathway analysis was performed on differentially expressed genes identified in the transcriptomic dataset. The results demonstrated that differentially expressed genes following 8-Cl-Ado treatment were primarily enriched in the PPAR signaling pathway, among others (Fig. S4A).

**Figure 7:**
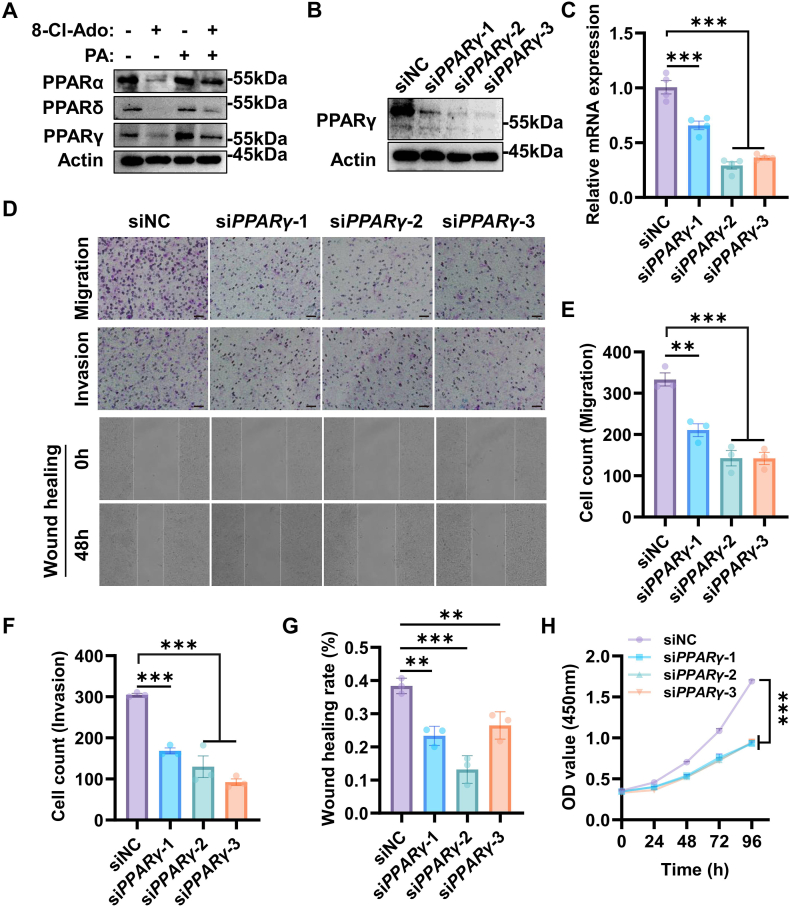
**PPARγparticipates in mediatingthe inhibitory effects of 8-Cl-Ado on lipid metabolism and liver cancer progression. (A)** Effect of 8-Cl-Ado on protein expression levels of PPAR isoforms (PPARα, PPARδ, PPARγ). **(B)** PPARγ protein expression levels in HepG2 cells following PPARγ knockdown. **(C)***PPARγ* mRNA expression levels in HepG2 cells following *PPARγ* knockdown. **(D)** Representative images showing the impact of *PPARγ* knockdown on migration, invasion, and wound healing capacities of HepG2 cells. Scale bar: 20 μm. **(E)** Quantification of *PPARγ* knockdown effects on cell migration. **(F)** Quantification of *PPARγ* knockdown effects on cell invasion. **(G)** Quantification of *PPARγ* knockdown effects on wound healing capacity. **(H)** Effect of *PPARγ* knockdown on OD450 nm absorbance in HepG2 cells with CCK-8 assay. All experiments were performed with at least three biologically independent replicates (*n* ≥ 3). Data were presented as mean ± standard error of the mean. Statistical significance was determined using Student's two-tailed *t*-test for two-group comparisons or one-way ANOVA for multiple groups. ^∗∗^*p* < 0.01, ^∗∗∗^*p* < 0.001.

To elucidate the specific mechanism by which 8-Cl-Ado inhibits lipid metabolism in HepG2 cells, the expression levels of three PPAR isoforms were measured.^35^^,^^36^ The data revealed that 8-Cl-Ado treatment reduced protein levels of all three PPAR isoforms and effectively suppressed palmitate-induced elevation of PPAR proteins (Fig. 7A). At the mRNA level, 8-Cl-Ado decreased *PPARα* and *PPARγ* expression and inhibited palmitate-induced up-regulation (Fig. S4B). These findings indicate that 8-Cl-Ado not only affects protein levels of all three PPAR isoforms but also influences PPARα and *PPARγ* expression at the transcriptional level, while showing no significant inhibitory effect on *PPARδ* gene expression. Critically, accumulating evidence highlights distinct functional roles for PPARα and PPARγ in lipid metabolism. PPARα primarily regulates fatty acid oxidation, whereas PPARγ serves as the master regulator of adipogenesis and lipid storage, playing a critical role in promoting lipid droplet formation and accumulation across various cell types, including hepatocytes and cancer cells.^36^^,^^37^^,^^42^ Given that experimental data from our study confirm the induction of lipid droplet formation, and considering PPARγ's well-established role as a key driver of lipid droplet biogenesis, we hypothesize that PPARγ may play a more pivotal role in mediating this process compared with PPARα. Efficient *PPARγ* knockdown in HepG2 cells (Fig. 7B and C) significantly impaired cell migration, invasion, wound healing (Fig. 7D–G), and proliferation (Fig. 7H) compared with control (siNC) groups. Collectively, these results establish *PPARγ* as the key molecular mediator through which 8-Cl-Ado regulates lipid metabolism and inhibits HCC progression.

### ADAR1 and PPARγ synergistically promote HCC progression via post-transcriptional regulation and correlate with poor prognosis

**Figure 8:**
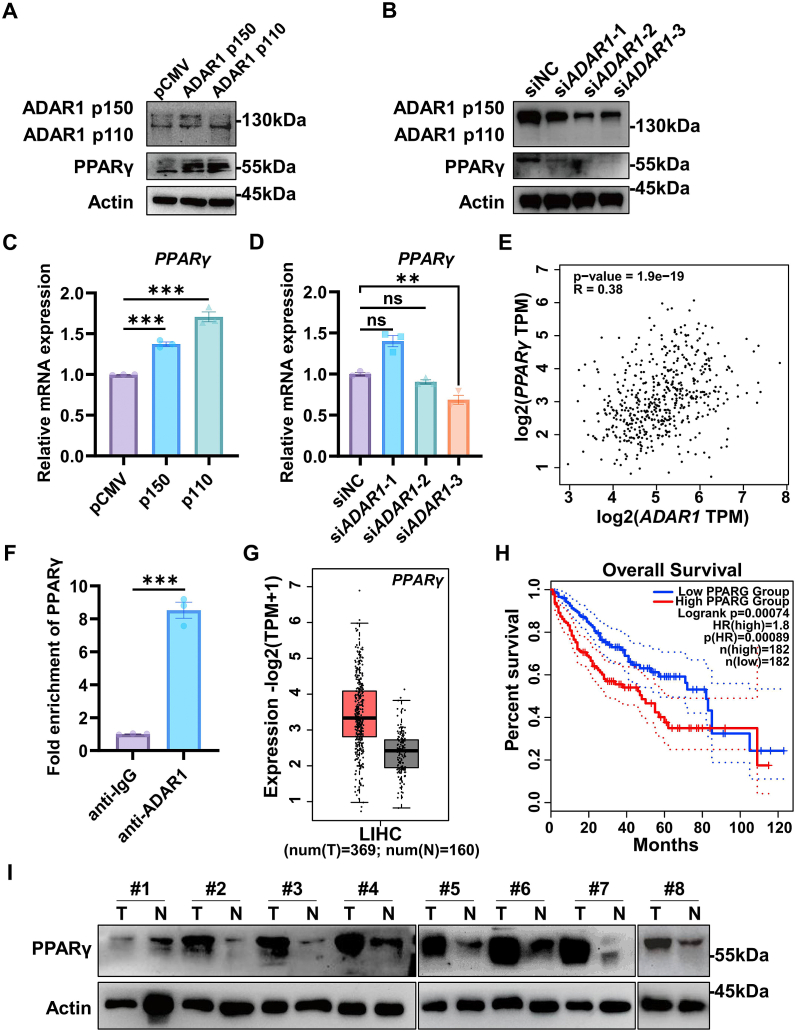
**ADAR1 promotes liver cancer progression via post-transcriptional regulation of PPARγ. (A)** PPARγ protein expression levels in HepG2 cells following ADAR1 overexpression. **(B)** PPARγ protein expression levels in HepG2 cells following *ADAR1* knockdown. **(C)***PPARγ* mRNA expression levels in HepG2 cells following *ADAR1* overexpression. **(D)***PPARγ* mRNA expression levels in HepG2 cells following *ADAR1* knockdown. **(E)** Correlation analysis between *ADAR1* and *PPARγ* expression (*r* = 0.38; GEPIA database). **(F)** RNA immunoprecipitation assay confirmed specific binding of ADAR1 protein to *PPARγ* mRNA. **(G)** Analysis of the GEPIA database demonstrating relative *PPARγ* levels in hepatocellular carcinoma (HCC) tumor tissues (red box, *n* = 369) versus normal liver tissues (gray box, *n* = 160). **(H)** Overall survival analysis of HCC patients stratified by PPARγ expression levels in the GEPIA database. **(I)** PPARγ protein expression levels in paired clinical HCC tumor tissues (T) and adjacent non-tumor tissues (N) (*n* = 8). All experiments were performed with at least three biologically independent replicates (*n* ≥ 3). Data were presented as mean ± standard error of the mean. Statistical significance was determined using Student's two-tailed *t*-test for two-group comparisons or one-way ANOVA for multiple groups. ^∗∗^*p* < 0.01, ^∗∗∗^*p* < 0.001.

ADAR1 overexpression significantly up-regulated both protein (Fig. 8A) and mRNA (Fig. 8C) levels of PPARγ. Conversely, ADAR1 knockdown markedly reduced PPARγ expression at both protein (Fig. 8B) and mRNA (Fig. 8D) levels. To investigate the regulatory relationship between ADAR1 and PPARγ, their correlation was analyzed. Analysis of the GEPIA database revealed a positive correlation between *ADAR1* and *PPARγ* expression (correlation coefficient *r* = 0.38; Fig. 8E). Given ADAR1's RNA-editing enzyme activity, RNA immunoprecipitation assay further confirmed that ADAR1 protein specifically bound to *PPARγ* mRNA (Fig. 8F). These findings suggest that ADAR1 regulates *PPARγ* expression at the post-transcriptional level.

Given that ADAR1 regulates PPARγ expression at the post-transcriptional level, the clinical significance of PPARγ was further investigated in HCC. Analysis of the GEPIA database revealed up-regulated PPARγ expression in HCC tissues compared with adjacent non-tumor tissues (Fig. 8G). Specifically, patients with high PPARγ expression showed significantly reduced survival relative to low-expression counterparts (Fig. 8H). Western blotting confirmed elevated PPARγ protein levels in HCC tissues (Fig. 8I), consistent with ADAR1-mediated regulatory mechanisms.

Taken together, these results demonstrate that ADAR1 and PPARγ synergistically promote HCC progression via post-transcriptional regulation and correlate with poor prognosis.

## Discussion

Metabolic reprogramming is a core driver of tumorigenesis and progression,^6^, ^7^, ^8^ with dysregulated lipid metabolism serving as a critical hallmark, particularly prominent in HCC.^8^^,^^9^ Lipids, including triglycerides, phospholipids, sphingolipids, and sterols, are not only essential structural components of cell membranes but also play vital roles in signal transduction and hormone synthesis.^43^ As the central organ for lipid metabolism, disruption of hepatic lipid metabolic pathways exerts a decisive influence on HCC progression. HCC cells show a high rate of *de novo* lipid synthesis, with multiple key enzymes involved in fatty acid synthesis being up-regulated and exhibiting enhanced activity in various cancers, including HCC; this correlates with poor clinical outcomes.^44^, ^45^, ^46^, ^47^ Our study demonstrates that PPARγ, which targets fatty acid and cholesterol synthesis, is significantly elevated in HCC compared with normal tissues, consistent with previous reports,^48^ suggesting that targeting lipid metabolic homeostasis imbalance may be an effective anti-HCC strategy. Distinct from prior studies revealing ADAR1-driven HCC progression through mechanisms such as regulating mitophagy,^49^ participating in non-coding RNA axes,^50^ or oxidative stress response pathways,^51^ this study is the first to elucidate that ADAR1 can bind to PPARγ mRNA, enhance lipid metabolism, and contribute to HCC progression.

8-Cl-Ado, an adenine nucleoside analog, exerts anti-cancer effects in tumors, such as leukemia,^26^, ^27^, ^28^ renal cell carcinoma,^30^ and breast cancer.^31^ We investigated 8-Cl-Ado in HCC, confirming its inhibitory effects and discovering that it reduces ADAR1 expression in a time- and concentration-dependent manner, suppresses the PPARγ signaling pathway, and reverses the malignant phenotype of liver cancer. While previous studies reported that 8-Cl-Ado inhibited breast cancer cell proliferation by suppressing ADAR1 to induce apoptosis,^52^ this study explored its anti-tumor effects in HCC and is the first to elucidate that 8-Cl-Ado regulates the PPARγ signaling pathway by down-regulating ADAR1. This discovery not only reveals the regulatory mechanism of the “ADAR1–PPARγ–lipid metabolism” axis in HCC but also provides a novel perspective for anti-tumor therapies targeting RNA editing enzymes.

ADAR1, an adenosine deaminase, is aberrantly overexpressed in multiple cancers and promotes malignant progression.^11^^,^^14^ Both ADAR1 isoforms, p150 and p110, contain three double-stranded RNA-binding domains (RBDs), a deaminase domain (DM), and a Zβ domain; notably, p150 includes a nuclear export signal (NES) within its N-terminal Zα domain.^13^ Through single-cell sequencing, clinical samples, and cellular models, this study validated the significant overexpression of ADAR1 in HCC tissues and confirmed its pro-tumorigenic role by enhancing the proliferation, migration, and invasion capabilities of HCC cells, aligning with previous research.^51^^,^^53^^,^^54^ We found that ADAR1 overexpression partially reversed the inhibitory effects of 8-Cl-Ado on the malignant phenotype, indicating that the anti-HCC effect of 8-Cl-Ado partially stems from its suppression of ADAR1. However, it is noteworthy that the clinical samples did not stratify HBV-positive and HBV-negative HCC subgroups, which may affect the generalizability of the conclusions. Concurrently, although the role of the double-stranded RBDs of ADAR1 has been reported in breast cancer,^52^ the structural basis for its specific recognition of PPARγ mRNA in HCC requires further elucidation.

A key innovation of this study is the first-time revelation of the molecular mechanism by which 8-Cl-Ado inhibits HCC by regulating lipid metabolism. Previous studies have reported that 8-Cl-Ado exerts anti-cancer activity by interfering with the cell cycle or inducing apoptosis/autophagy,^29^^,^^55^ but its regulation of lipid metabolism in liver cancer remains unelucidated. Transcriptomic analysis revealed that 8-Cl-Ado suppressed the expression of key genes involved in cholesterol synthesis and fatty acid metabolism (*e.g.*, FASN, SCD), and reduced intracellular lipid droplet accumulation and lipid content. Follow-up validation experiments further demonstrated that while 8-Cl-Ado down-regulated protein levels of all PPAR isoforms, it significantly inhibited the transcription of both *PPARα* and *PPARγ* at the mRNA level, whereas *PPARδ* remained relatively unaffected. Considering the distinct functional roles of PPARα and PPARγ, and the observation that silencing *PPARγ* effectively inhibited HCC cell proliferation and metastasis, these findings strongly suggest that PPARγ is the central node mediating 8-Cl-Ado's lipid metabolic regulation. Clinical data analysis also strengthens the evidence for PPARγ as a negative prognostic indicator in HCC.

Intriguingly, PPARγ appears to play two seemingly opposing roles in HCC. On one hand, PPARγ activation can inhibit cell proliferation and growth based on its differentiation-inducing capacity.^56^ On the other hand, PPARγ activation promotes glycolysis in cells, thereby enhancing HCC proliferation, migration, and invasion.^39^ Given this dual role of PPARγ as both an oncogene and tumor suppressor, we speculate that PPARγ may function as a stress-adaptive regulatory node in HCC progression. In response to specific cellular stresses, such as metabolic remodeling, oxidative stress, and replicative stress, PPARγ orchestrates tailored regulatory responses to alleviate stress and efficiently acquire energy sources. This study revealed a novel regulatory relationship between ADAR1 and PPARγ: their expression was positively correlated, and ADAR1 interacted with PPARγ. This discovery advances the understanding of the lipid metabolic regulatory network in tumors. However, whether ADAR1 regulates PPARγ via editing-dependent or editing-independent mechanisms (*e.g.*, functioning as an RNA-binding protein), and its interaction mechanisms with other lipid metabolism-related transcripts, require further investigation. Additionally, one study reported that ADAR1 overexpression reduced PPARγ expression,^57^ contradicting our results. This discrepancy may stem from distinct biological mechanisms: During adipogenesis, ADAR1 binds to Dicer to enhance miR-155-5p production, thereby inhibiting the C/EBPβ-PPARγ cascade and limiting excessive fat accumulation^57^; conversely, in the HCC microenvironment, ADAR1 binds to PPARγ mRNA to drive lipid synthesis, fueling proliferation. Furthermore, the two major PPARγ isoforms, γ1 and γ2, differ in tissue distribution and responsiveness to developmental and nutritional signals.^58^, ^59^, ^60^ ADAR1 might achieve specific outputs by selectively binding to different isoform mRNAs. ADAR1 can function via its editing activity or protein–protein interactions, or as an RNA-binding protein.^12^ While we demonstrated ADAR1 binding to PPARγ mRNA, the precise regulatory mechanism remains unclear and warrants further exploration.

In conclusion, our findings elucidate that 8-Cl-Ado suppresses lipid metabolism by targeting the ADAR1–PPARγ axis, thereby impeding the progression of HCC. This pathway provides a novel target and theoretical foundation for HCC treatment based on metabolic intervention, potentially playing a critical role in inhibiting HCC progression. Although further studies are needed to clarify the precise regulatory mechanism underlying the binding of ADAR1 to *PPARγ* mRNA, our research offers new insights into the molecular mechanism by which 8-Cl-Ado inhibits HCC. Further deciphering the ADAR1-mediated metabolic-epigenetic regulatory network will advance the development of precision therapy strategies for HCC.

## CRediT authorship contribution statement

**Jing Liu:** Writing – original draft, Validation, Software, Methodology, Investigation, Funding acquisition, Formal analysis, Data curation, Conceptualization. **Yongle Zhao:** Writing – original draft, Validation, Software, Methodology, Formal analysis, Data curation. **Xue Gong:** Writing – original draft, Validation, Software, Methodology, Formal analysis, Data curation, Conceptualization. **Lin Yuan:** Supervision, Data curation. **Shengyong Yang:** Software, Methodology, Data curation. **Wenwen Jian:** Software, Formal analysis, Data curation. **Han Yan:** Software, Formal analysis, Data curation. **Honglin Chen:** Visualization, Methodology, Investigation, Data curation. **Zhicheng Yang:** Visualization, Formal analysis, Data curation. **Yiheng Sun:** Software, Methodology, Investigation. **Tianle Gu:** Software, Formal analysis, Data curation. **He Lu:** Methodology, Formal analysis, Data curation. **Hongyun Zhao:** Writing – review & editing, Writing – original draft, Validation, Supervision, Resources, Funding acquisition, Data curation, Conceptualization. **Zeng Tu:** Writing – review & editing, Writing – original draft, Validation, Supervision, Resources, Project administration, Methodology, Investigation, Funding acquisition, Data curation, Conceptualization.

## Ethics declaration

This study involves human participants and was approved by the Ethics Committee of the Second Affiliated Hospital of Chongqing Medical University (Approval No. 202441). Written informed consent was obtained from all patients.

## Data availability

All data generated or analyzed in this study are included in the article, and further inquiries can be directed to the corresponding author.

## Funding

This work was supported in part by grants from the General Program of the Chongqing Natural Science Foundation (China) (No. CSTB2022NSCQ-MSX0909 to H.Z., CSTC2021JCYJ-MSXMX0158 to Z.T., CSTB2024NSCQ-MSX0179 to J.L.), the Kuanren Talents Program of the Second Affiliated Hospital of Chongqing Medical University (China) (No. 2021240308 to H.Z.), and the Group Medical Aid Project for the Tibet Autonomous Region, supported by the Natural Science Foundation of the Tibet Autonomous Region of China (No. XZ2023ZR-ZY75(Z) to H.Z.).

## Conflict of interests

The authors declared that the research was conducted in the absence of any commercial or financial relationships that could be construed as a potential conflict of interests.
