# Supplementary material for: 8-Chloroadenosine suppresses hepatocellular carcinoma progression via ADAR1/PPARγ axis-mediated lipid metabolism
Source: Genes Dis. 2025 Sep 26;13(5):101874. doi: 10.1016/j.gendis.2025.101874 (PMC13276139; doi:10.1016/j.gendis.2025.101874)
Supplement: Multimedia component 1 [file mmc1.docx]

**Figure S1 Validation of ADAR1 overexpression in liver cancer cells. (A)** *ADAR1* mRNA expression levels in HepG2 cells following ADAR1 overexpression. **(B)** *ADAR1* mRNA expression levels in Huh7 cells following ADAR1 overexpression. **(C)** ADAR1 protein expression levels in HepG2 cells following ADAR1 overexpression. **(D)** ADAR1 protein expression levels in Huh7 cells following ADAR1 overexpression. All experiments were performed with at least three biologically independent replicates (*n* ≥ 3). Data were presented as mean ± standard error of the mean. Statistical significance was determined using Student's two-tailed *t*-test for two-group comparisons or one-way ANOVA for multiple groups. ^*^*P* < 0.05, ^**^*P* < 0.01, ^***^*P* < 0.001.

**Figure S2 ADAR1 overexpression promotes proliferation, migration, and invasion of liver cancer cells.** **(A)** Representative images showing the impact of ADAR1 overexpression on migration, invasion, and wound healing capacities of HepG2 and Huh7 cells. Scale bar: 20 μm. **(B)** Quantification of ADAR1 overexpression effects on cell migration in HepG2 and Huh7 cells. **(C)** Quantification of ADAR1 overexpression effects on cell invasion in HepG2 and Huh7 cells. **(D)** Quantification of ADAR1 overexpression effects on wound healing capacity in HepG2 and Huh7 cells. **(E)** Effect of ADAR1 overexpression on OD_450_ in HepG2 and Huh7 cells with CCK-8 assay. All experiments were performed with at least three biologically independent replicates (*n* ≥ 3). Data were presented as mean ± standard error of the mean. Statistical significance was determined using Student's two-tailed *t*-test for two-group comparisons or one-way ANOVA for multiple groups. ^**^*P* < 0.01, ^***^*P* < 0.001.

**Figure S3 ADAR1 overexpression counteracts 8-Cl-Ado-mediated inhibition of proliferation, migration, and invasion of liver cancer cells. (A)** Representative images showing the effects of 8-Cl-Ado treatment modulation via ADAR1 overexpression on migration, invasion, and wound healing capacities of HepG2 cells. All experiments were performed with at least three biologically independent replicates (*n* ≥ 3). Scale bar: 20 μm. **(B)** Representative images showing the effects of 8-Cl-Ado treatment modulation via ADAR1 overexpression on migration, invasion, and wound healing capacities of Huh7 cells. All experiments were performed with at least three biologically independent replicates (*n* ≥ 3). Scale bar: 20 μm. (C) The heatmap visualizing the expression patterns and significance (Log₂(fold change)) of genes associated with cholesterol biosynthesis and fatty acid metabolism in 8-Cl-Ado-treated HepG2 and Huh7 cells.

**Figure S4 KEGG enrichment analysis and PPAR isoform expression profiles. (A)** KEGG pathway enrichment analysis of differentially expressed genes in 8-Cl-Ado-treated HepG2 cells. The *PPAR* signaling pathway is highlighted. **(B)** Effect of 8-Cl-Ado on mRNA expression levels of *PPAR* isoforms (*PPARα*, *PPARδ*, *PPARγ*) in HepG2 cells. This experiment was performed with at least three biologically independent replicates (*n* ≥ 3). Data were presented as mean ± standard error of the mean. Statistical significance was determined using Student's two-tailed *t*-test for two-group comparisons or one-way ANOVA for multiple groups. ^*^*P* < 0.05, ^**^*P* < 0.01, ^***^*P* < 0.001.
