# Supplementary material for: 8-Chloroadenosine suppresses hepatocellular carcinoma progression via ADAR1/PPARγ axis-mediated lipid metabolism
Source: Genes Dis. 2025 Sep 26;13(5):101874. doi: 10.1016/j.gendis.2025.101874 (PMC13276139; doi:10.1016/j.gendis.2025.101874)
Supplement: Multimedia component 2 [file mmc2.pdf]

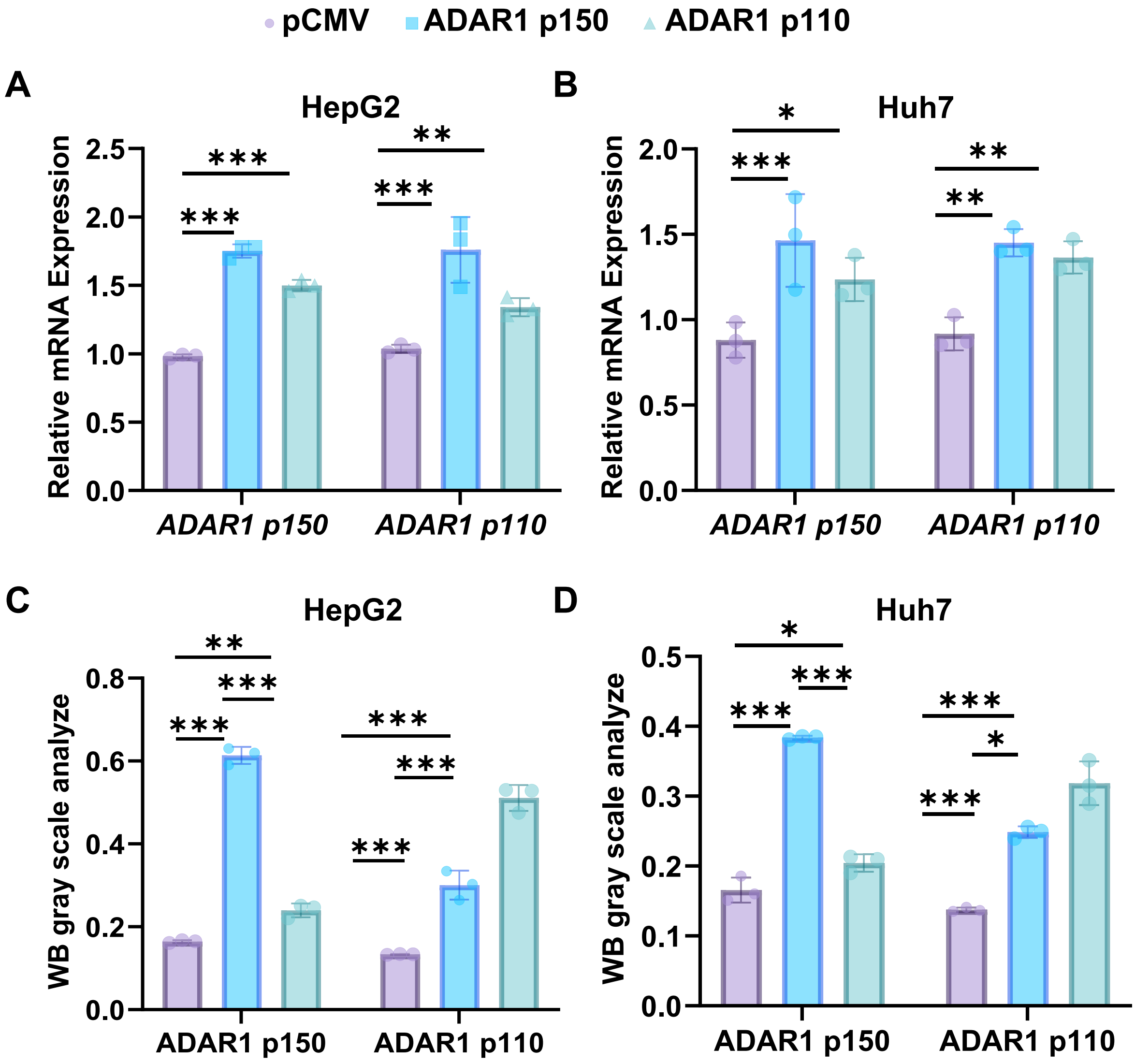

Supplementary Figure 1

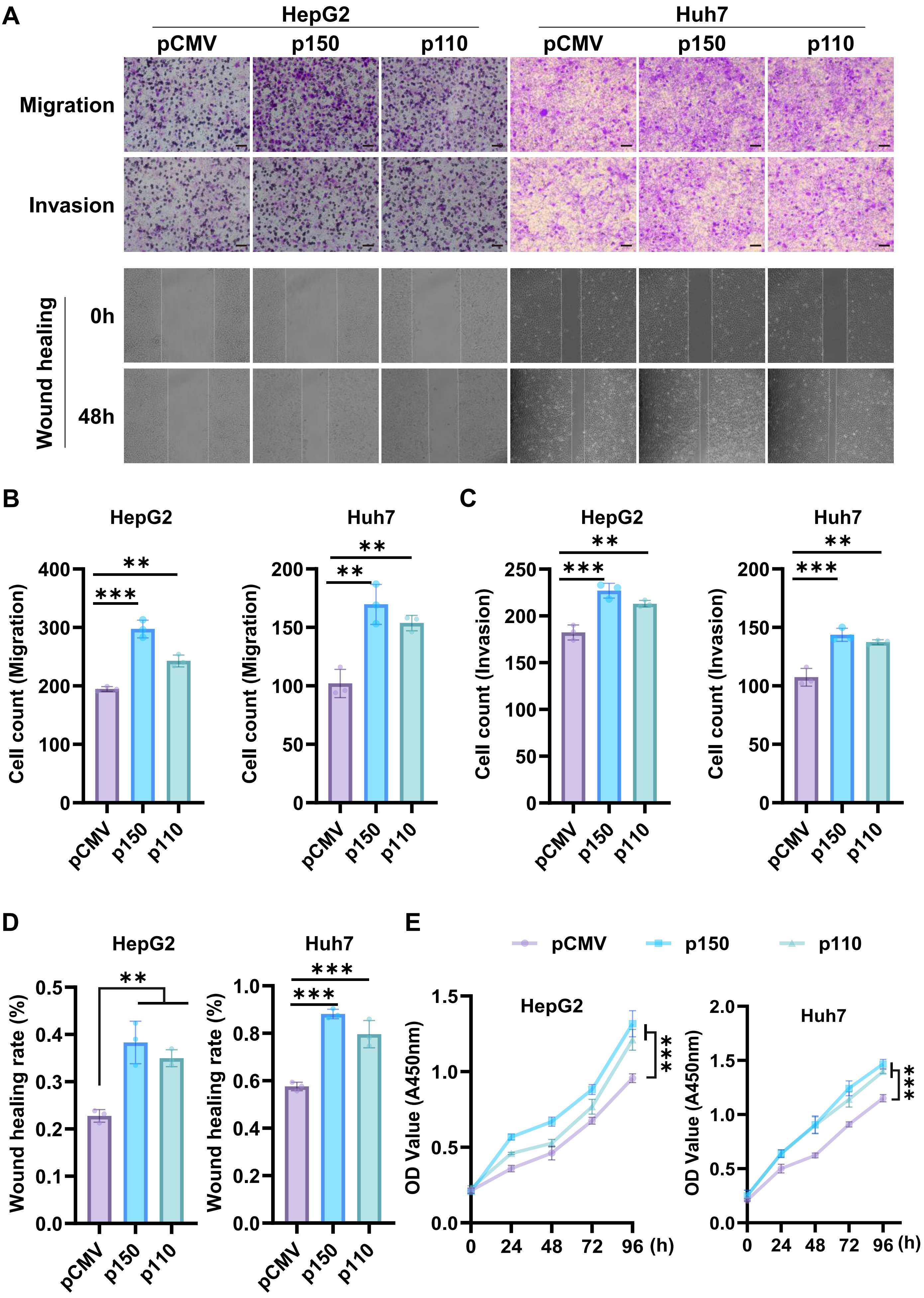

Supplementary Figure2

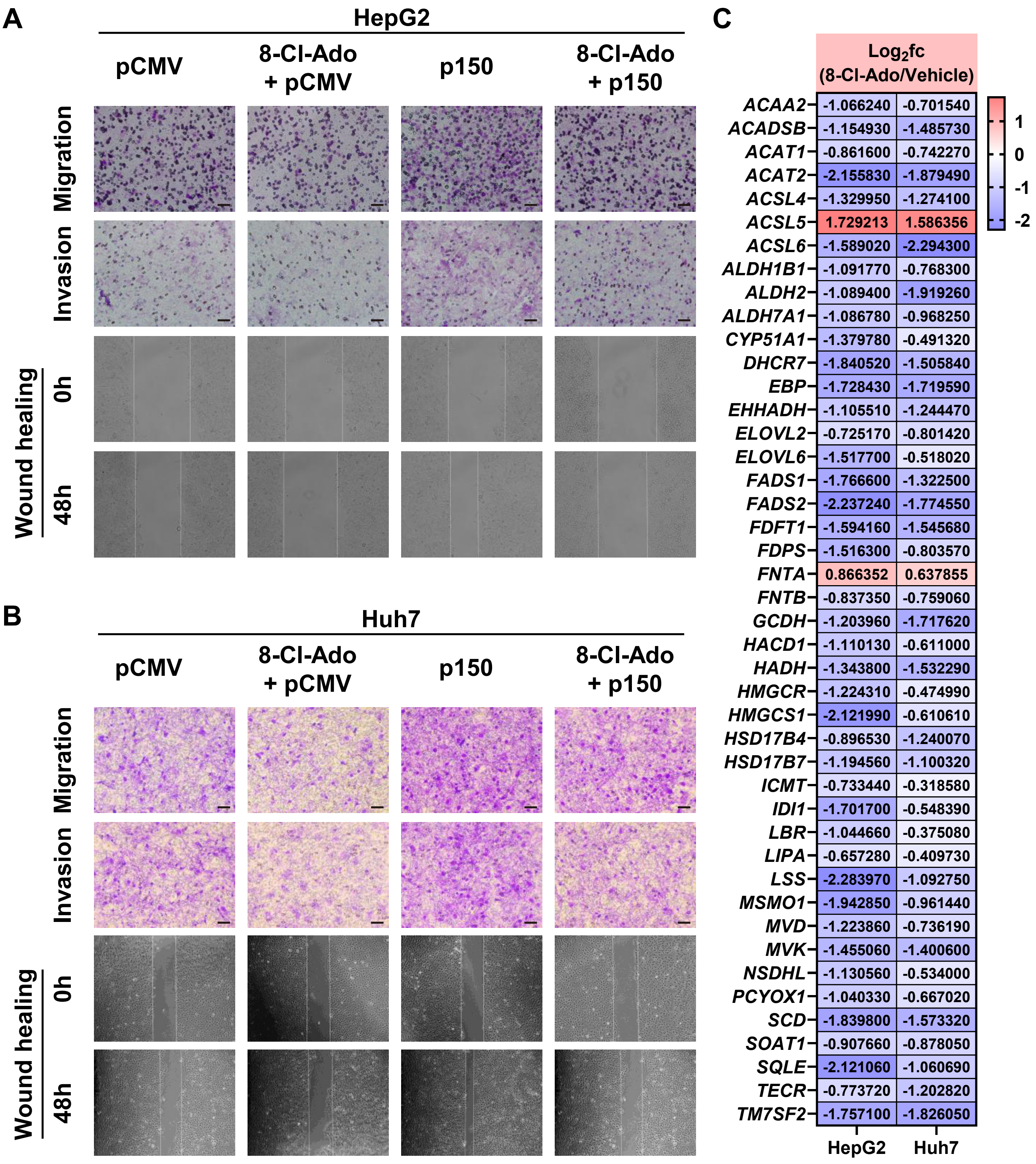

Supplementary Figure 3

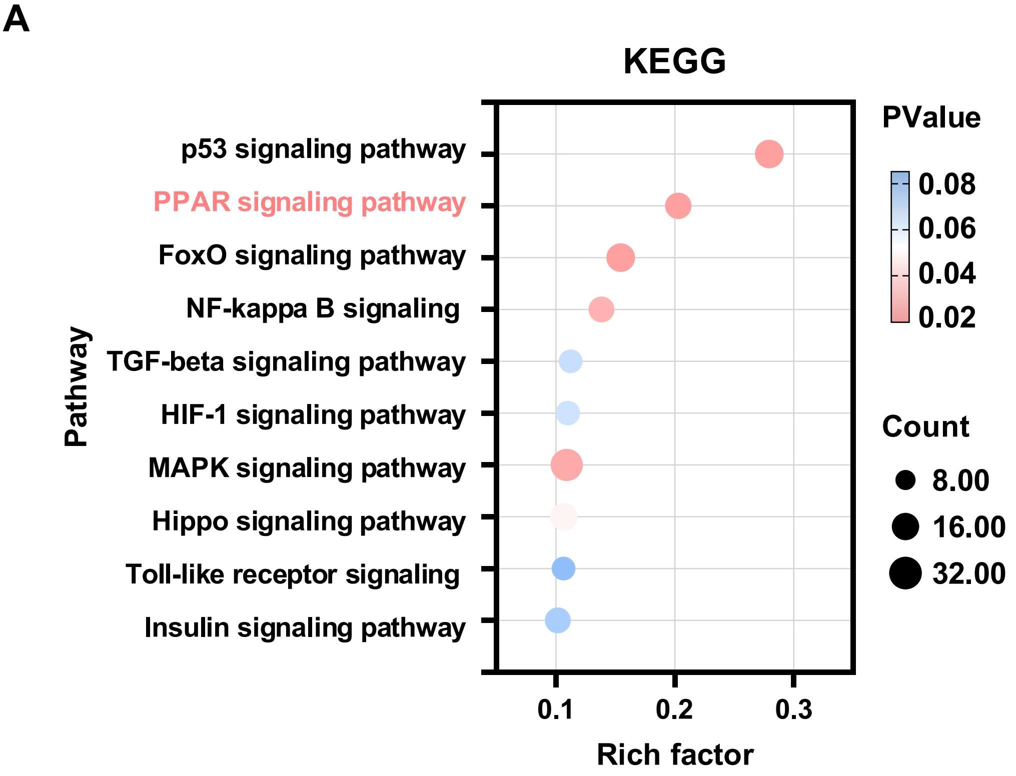

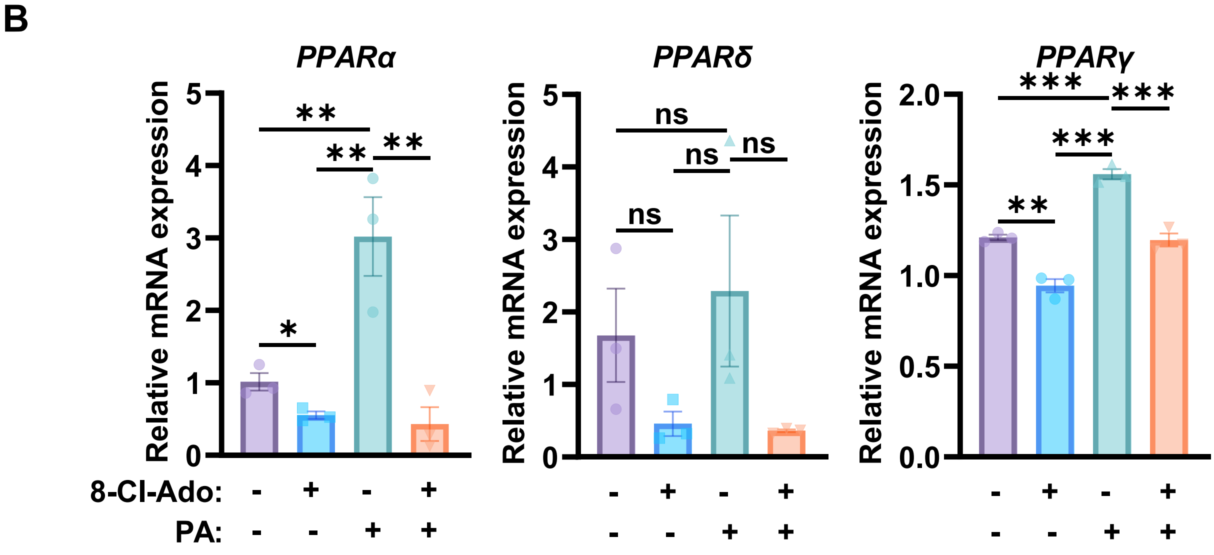

Supplementary Figure 4
